# Highly Porous Yet Transparent Mechanically Flexible Aerogels Realizing Solar-Thermal Regulatory Cooling

**DOI:** 10.1007/s40820-024-01356-x

**Published:** 2024-02-26

**Authors:** Meng Lian, Wei Ding, Song Liu, Yufeng Wang, Tianyi Zhu, Yue-E. Miao, Chao Zhang, Tianxi Liu

**Affiliations:** 1 State Key Laboratory for Modification of Chemical Fibers and Polymer Materials, College of Materials Science and Engineering, Donghua University, Shanghai, 201620 People’s Republic of China; 2 Key Laboratory of Synthetic and Biological Colloids, Ministry of Education, School of Chemical and Material Engineering, Jiangnan University, Wuxi, 214122 People’s Republic of China

**Keywords:** Transparent aerogel, Cellulose nanofiber aerogel, Delaminated gelation, Thermal insulation, Passive daytime radiative cooling

## Abstract

The demand for highly porous yet transparent aerogels with mechanical flexibility and solar-thermal dual-regulation for energy-saving windows is significant but challenging. Herein, a delaminated aerogel film (DAF) is fabricated through filtration-induced delaminated gelation and ambient drying. The delaminated gelation process involves the assembly of fluorinated cellulose nanofiber (FCNF) at the solid–liquid interface between the filter and the filtrate during filtration, resulting in the formation of lamellar FCNF hydrogels with strong intra-plane and weak interlayer hydrogen bonding. By exchanging the solvents from water to hexane, the hydrogen bonding in the FCNF hydrogel is further enhanced, enabling the formation of the DAF with intra-layer mesopores upon ambient drying. The resulting aerogel film is lightweight and ultra-flexible, which possesses desirable properties of high visible-light transmittance (91.0%), low thermal conductivity (33 mW m^−1^ K^−1^), and high atmospheric-window emissivity (90.1%). Furthermore, the DAF exhibits reduced surface energy and exceptional hydrophobicity due to the presence of fluorine-containing groups, enhancing its durability and UV resistance. Consequently, the DAF has demonstrated its potential as solar-thermal regulatory cooling window materials capable of simultaneously providing indoor lighting, thermal insulation, and daytime radiative cooling under direct sunlight. Significantly, the enclosed space protected by the DAF exhibits a temperature reduction of 2.6 °C compared to that shielded by conventional architectural glass.

**Supplementary Information:**

The online version contains supplementary material available at 10.1007/s40820-024-01356-x.

## Highlights

- A lamellar-structured fluorinated cellulose nanofiber aerogel film is prepared by filtration-induced delaminated gelation and ambient drying.
- The aerogel film demonstrates exceptional mechanical flexibility and resistance to complex deformations.
- The aerogel film displays low thermal conductivity, high visible-light transmittance and superior selective infrared emissivity, rendering it high solar-thermal regulatory cooling performance.

**Supplementary Information:**

The online version contains supplementary material available at 10.1007/s40820-024-01356-x.

## Graphical abstract

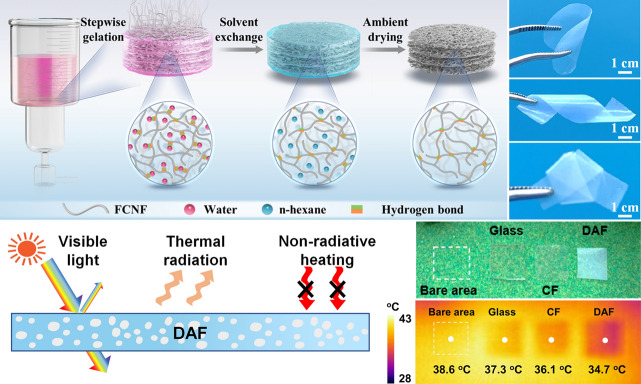

## Introduction

Buildings account for nearly 40% of global energy consumption with a significant portion dedicated to refrigeration systems specifically air conditioners, resulting in substantial environmental and energy consequences [1]. To create an energy-efficient and comfortable indoor living environment, it is crucial to minimize heat exchange between the indoors and outdoors by reducing heat conduction, convection, and radiation [2–4]. Among the various components of building envelopes, windows are the least energy-efficient, contributing to approximately 40%–60% of total energy usage [5]. An ideal energy-saving window material should possess high thermal-insulating properties and effectively modulate solar radiation. Thermal insulation helps prevent unwanted heat transfer from the outside to the inside, while solar-radiation modulation facilitates efficient indoor lighting and enables effective control of infrared emissions. These features are necessary to achieve solar-thermal regulatory cooling without excessive energy consumption [6, 7]. Passive daytime radiative cooling technology has gained attention in recent years as it dissipates heat by reflecting sunlight and transferring heat through the atmospheric transparent window to cold outer space without any additional energy consumption [8–13]. However, achieving high solar-thermal regulatory cooling using existing energy-saving windows presents a challenge [14, 15]. Therefore, there is a critical need to develop visible-light transparent and thermally insulating materials capable of efficient solar-thermal regulatory cooling for the design of next-generation energy-saving windows. However, this development faces significant challenges.

Aerogel is a nanoscale porous solid formed by replacing the liquid phase of a gel with gas through a specific drying technique [16–22]. It possesses several remarkable properties such as low density, high porosity, low thermal conductivity, and controllable nanoporous structure, making it a promising material for efficient heat transfer regulation in window applications [9, 23–29]. However, there are challenges associated with the utilization of aerogels, including poor mechanical properties, large pore sizes leading to optical opaque, and surfaces rich in hydrophilic functional groups, which limit their applications in hot and humid environments [30–35]. Cellulose nanofibers (CNFs), derived from cellulose, are filamentary fiber materials with diameters ranging from 5 to 20 nm and lengths ranging from several hundred nanometers to tens of micrometers [36–40]. These CNFs can be intertwined to form a nanofiber aerogel, which offers highly customizable pore structures and exceptional mechanical properties [41–44]. Additionally, CNFs contain abundant C–O–C and C–OH groups within their molecular chains, which exhibit strong absorption in the atmospheric transparency window and hold the potential for efficient radiative thermal regulation [45, 46]. Transparent radiative coolers and aerogel coolers based on cellulose materials with high reflectance and thermal insulation have been developed for cooling applications; however, they often exhibit either poor thermal insulation performance or limited visible light transmittance [47–49]. Therefore, the development of CNF-based aerogels with high mechanical flexibility, solar-thermal dual modulation capabilities, and hydrophobic properties holds great significance for constructing energy-saving windows but is challenging.

Herein, we present the preparation of delaminated aerogel film (DAF) by the filtration-induced delaminated gelation and ambient drying strategy. The fabrication strategy involves three steps including delaminated gelation of fluorinated CNF (FCNF), solvent exchange of water with hexane and ambient pressure drying. The conventional filtration of the aqueous suspension of FCNF leads to the formation of a unique nanofiber hydrogel at the filter-suspension interface, and the gelation is induced by the strong intra-plane hydrogen bonding between individual FCNF. Subsequent solvent exchange and ambient drying yield the DAF with a delaminated stacked structure with intra-plane mesopores. The high-density hydrogen bonding within the intra-layer nanofibers and between the delaminated stacked structure imparts the DAF with exceptional mechanical flexibility and resistance to complex deformations. The aerogel film also exhibits a low thermal conductivity of 33 mW m^−1^ K^−1^ and a high visible-light transmittance of 91.0% due to the presence of mesopores within the aerogel films. Notably, the DAF demonstrates a high selective infrared emissivity of 90.1% in the atmospheric transparency window, owing to the enriched chemical groups of C–O–C, Si–O-Si, Si–O-C, and C-F. Importantly, the aerogel film shows high hydrophobicity and excellent durability in complex outdoor high-temperature and humidity environments, which are attributed to the presence of abundant fluorine-containing groups with lower surface energy and resistance to UV irradiation. As a demonstration, the DAF could act as a thermo-optic dual-regulated energy-saving window material ensuring efficient indoor lighting and indoor radiative thermoregulation, which effectively prevents heat migration from the outdoor environment to the indoors. Compared to traditional architectural glass, the DAF achieves an indoor cooling effect with a temperature drop of approximately 2.6 °C while maintaining high visible-light transmittance under direct sunlight. This study might provide a novel concept for constructing optically transparent and radiative cooling aerogel films that possess high mechanical flexibility and advanced solar-thermal regulating capabilities.

## Experimental Section

### Materials

A powder sample of CNF was purchased from Guilin Odd Macro Technologies (China). Perfluorodecyltriethoxysilane (PFOTES, 97%) was purchased from Adamas Reagent Co. (China). Ethanol, propanol (AR, > 99.5%) and hexane (AR, > 97.0%) were purchased from Titan Reagent Co. (China). Deionized water was used throughout the experiments. All the chemicals were used without further purification.

### Preparation of FCNF

A predetermined amount of PFOTES (1 wt%) was added into a mixed solution of ethanol and water (9/1, vol/vol) with a volume of 100 mL and then stirred for 3 h at room temperature to achieve partial hydrolysis of silane. Next, 1 g of CNF powder was added to the resulting dispersion and ultrasonicated for 5 min. The mixed suspension was then stirred at 70 °C for 6 h followed by washing with ethanol three times. Finally, the FCNF was obtained by drying the suspension at 40 °C overnight.

### Preparation of the DAF, Compact film (CF) and Casting Aerogel Film (CAF)

A homogeneous dispersion of 0.2 g of FCNF in 20 mL of water was prepared and subsequently vacuum-filtered through a 0.45 µm pore-size PVDF membrane (GVWP, Millipore). The filtration process continued until gelation on the surface of the filter paper was fully achieved. The resultant hydrogel film was then transferred into a Petri dish full with propanol and soaked for 2 h. After replacing the water in the hydrogel film with propanol, the solvent exchange process was repeated by using hexane as the exchange solvent. Afterward, the solvent-exchanged film was dried at room temperature and ambiently dried for 2 h to obtain the DAF. A filtrated CNF aerogel film was prepared using a similar fabrication strategy as the DAF, with the only difference being the substitution of FCNF with CNF for comparative purposes. Additionally, a comparison sample of the filtrated FCNF hydrogel film was directly dried at room temperature and ambient pressure to obtain the CF. The CAF with similar solid content as the DAF was prepared by solution casting of the FCNF dispersion followed by a similar solvent exchange and ambient drying process as the DAF.

### Materials Characterization

Chemical compositions of CNF and FCNF were determined by Fourier transform infrared spectroscopy (FTIR, Nicolet 570, USA) in the wavelength range of 4000–400 cm^−1^. Elements in CNF and FCNF were characterized by X-ray photoelectron spectroscopy (S1703706). Mid-IR spectral emissivity was measured by an FT-IR spectrometer (Nicolet iS50, Thermo Fisher Scientific) equipped with a gold-coated integrating sphere via the reflectance measurement method at room temperature. Rheological behaviors of FCNF hydrogel films were measured by a HAAKE MARS rheometer. Morphologies and microstructures of powder and film samples were characterized using field-emissions scanning electron microscopy (FESEM, Ultra 55). Brunauer–Emmett–Teller (BET) surface areas were determined by a nitrogen physisorption using a Quantachrome Autosorb-iQ-AG porosimeter. Pore size distributions were calculated from the adsorption isotherm according to the nonlocal density functional theory (NLDFT) equilibrium model method for slit pores provided by Quantachrome. Mechanical properties of film samples were measured using an Instron Universal Testing Machine (Model 5567). For the tensile measurements, the strip sample in the sizes of 10 mm × 8 mm × 100 µm was stretched at a tensile rate of 10 mm min^−1^. Small-angle X-ray scattering (SAXS) was carried out on a SAXSessmc2 X-ray scattering diffraction. X-ray diffraction (XRD) analysis was carried out on a Rigaku D/max-2550 PC X-ray diffractometer with Cu K_α_ radiation. PerkinElmer Lambda 950 VU-visible-NIR absorption spectrophotometer with an integrating sphere was used to record the transmission spectra of the DAF, CAF and CF in the UV–visible-NIR range. Thermal conductivity was detected on a hot disk thermal analyzer (Hot Disk TPS 2500S, Sweden) according to the transient plane source method (IOS 22007–2:2015). Thermographic images were recorded by an infrared thermal camera (FOTRIC220S, China). Contact angles were measured by a contact angle analyzer (OCA40 Micra, China).

## Results and Discussion

### Preparation, Flexibility and Microstructures of the DAF

**Fig. 1:**
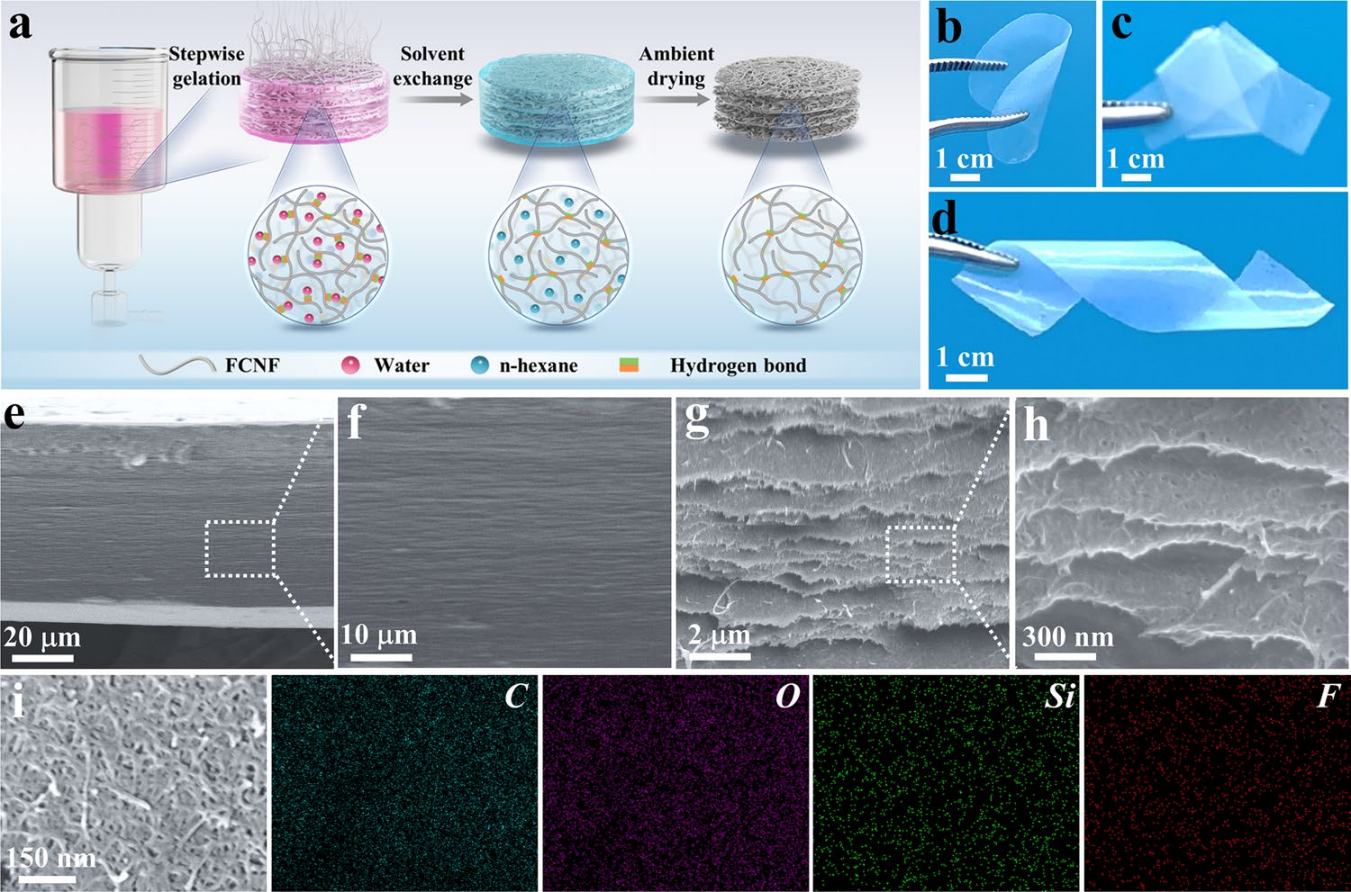
Preparation, flexibility and microstructures of the DAF. **a** Schematic of the fabrication procedure of DAF. **b–d** Photographs of DAF under, bending, knotting and twisting. Cross section FESEM images of DAF **e, f** before and **g, h** after tearing. **i** SEM image and element mappings (C, O, Si, F) of DAF

Figure 1a presents the schematic of the fabrication procedure of DAF using the filtration-induced delaminated gelation and ambient drying strategy. An aqueous dispersion of FCNF was utilized as the filtrate, and the vacuum during filtration established a negative pressure, facilitating intermolecular interaction of FCNF through hydrogen bonding. This resulted in the formation of a unique nanofiber-assembled ultrathin layered hydrogel at the interface between the filter paper and the above dispersion. The delaminated gelation process was confirmed by the observation of the formation of a gradient porous structure in the freeze-dried FFCNF hydrogel intermediate during the filtration process (Fig. S1). Rheological tests of the as-obtained FFCNF hydrogel show that the elastic modulus (*G'*) of the hydrogel is much higher than the corresponding loss modulus (*G"*) at the small strain and decreases at a relatively high oscillatory (Fig. S2), clearly indicating the formation of the FFCNF hydrogel during the filtration. Following solvent exchange and ambient drying, the resulting DAF exhibited the delaminated stacked structure with intra-layer mesopores, achieving an impressive porosity of 98.2%. This is because the used FCNF has high aspect ratios that could be interconnected to form a layer-by-layer lamellar structure with strong intra-plane hydrogen bonding and relatively weak interlayer hydrogen bonding. After the exchange of solvents from water to hexane, the thickness of the hydrogel significantly decreases (Fig. S3), indicating that the interlayer hydrogen bonding of DAF was enhanced. As a consequence, the unique DAF with a delaminated stacked and intra-layer nanopore structure is prone to form after solvent exchange and ambient drying. Compared with the casting aerogel film, the DAF is capable of maintaining the high structural stability of the delaminated structure after the removal of non-polar solvents in an ambient condition ascribing to the presence of high-density intralayer hydrogen bonds. The strong intermolecular hydrogen bonding between the nanofibers enabled the DAF to withstand complex deformations such as bending, knotting, and twisting without fractures (Fig. 1b-d). Moreover, the DAF could be easily tailored into desired shapes (Fig. S4).

Microstructural analysis using field emission scanning electron microscope (FESEM) revealed an oriented layered structure in the cross section SEM images of DAF due to the delaminated stacking of the nanofibers during gelation (Fig. 1e, f). Cross section FESEM images of intentionally ripped DAF samples revealed the presence of abundant nanosized pores embedded within their lamellar architecture (Fig. 1g, h). These nanopores were formed as a result of the stabilized porous structure due to high-density hydrogen bonding within layers. Scanning electron microscopy and electron dispersive spectroscopy (SEM–EDS) mappings confirmed the successful fluorination of CNF through a simple solution silanization reaction (Fig. 1i), supported by Fourier transform infrared (FTIR) spectroscopy and X-ray photoelectron spectroscopy (XPS) results (Figs. S5 and S6). The presence of fluorine-containing functional groups with low surface energy among the FCNF facilitated surface enrichment, thereby preserving the mesopores within the FCNF aerogel structure during solvent exchange and ambient drying. Furthermore, the low-surface-energy characteristics of FCNF effectively mitigated the aerogel structure shrinkage by minimizing capillary forces during ambient drying.

### Nanopore Formation Mechanism of the DAF

**Fig. 2:**
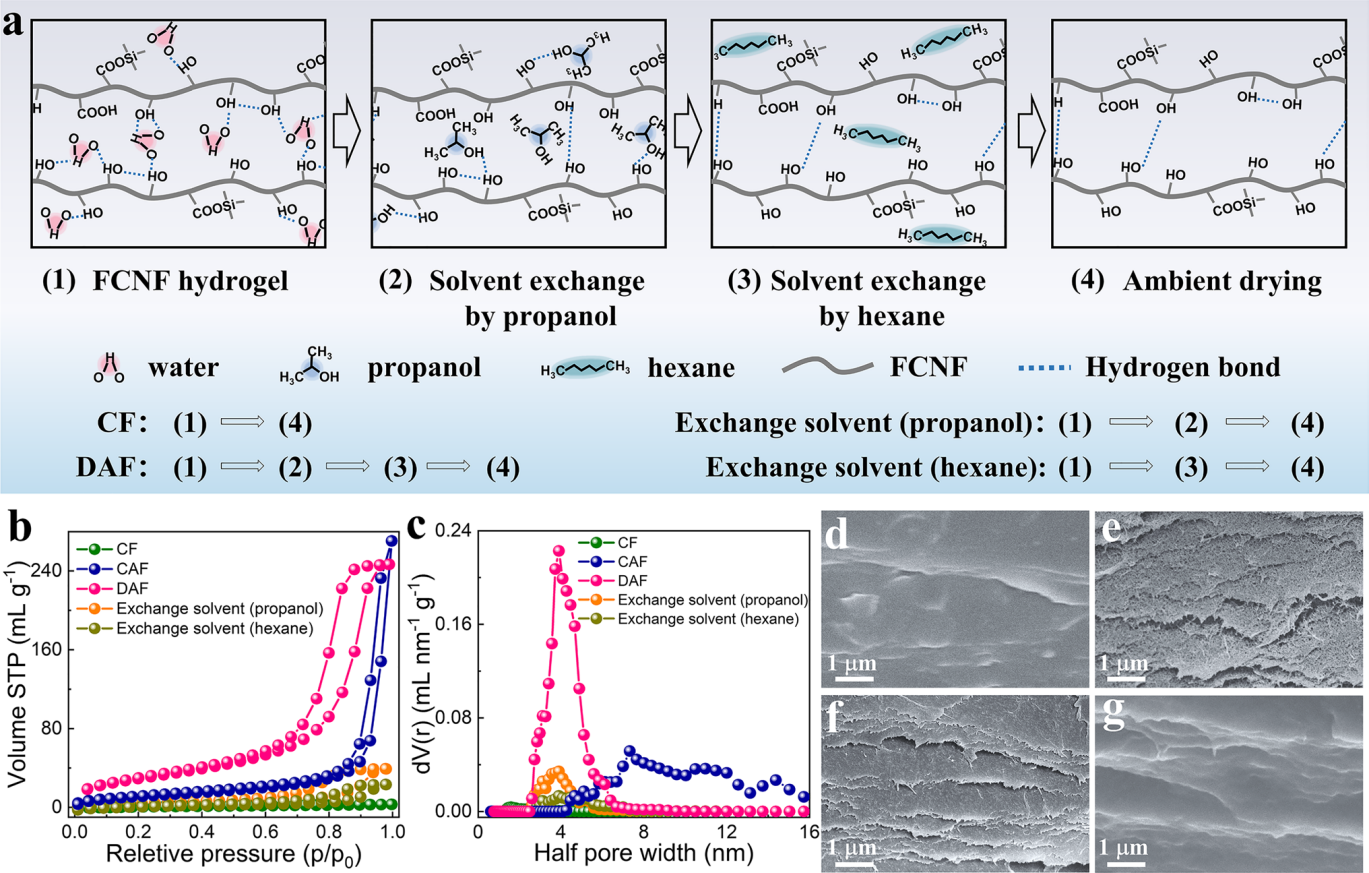
Pore-forming mechanism of the DAF. **a** Schematic of the various procedures for the water removals in FFCNF hydrogel. **b, c** Nitrogen adsorption/desorption isotherms and pore size distributions of CF, CAF, DAF and samples by solvent exchange with propanol and hexane, respectively. **d–g** FESEM images of the torn locations of CF, CAF and samples by solvent exchange with propanol and hexane, respectively

The structural evolution of mesopores of DAF during the solvent replacement and ambient drying processes was further investigated. As illustrated in Fig. 2a, the DAF was ambient dried after being solvent-exchanged with propanol and hexane, whereas the filtrated FCNF compact film (CF) was ambient dried directly from the FFCNF hydrogel. The samples with the exchange solvents of propanol and hexane were also produced to investigate the impact of the surface energy of solvents on the nanopore structures of the resultant aerogel films. The majority of hydrogel bonds in a typical FFCNF hydrogel are formed between the hydroxyl groups of FCNF and water. The hydrogen bonding between the FCNF and the exchanged solvent was attenuated when the solvent surface energy of the FFCNF hydrogel decreased after the solvent exchange with propanol. The contact between the FCNF and the surrounding solvents was further decreased by the solvent exchange with hexane, resulting in the strengthening of interfacial interactions between the individual FCNF. As a sequence, the nanoporous structures of the FCNF structures were successfully retained through the subsequent ambient drying. This illustrates that pairing the low-surface-energy surfaced FCNF with surrounding solvents circumvents the highly porous aerogel structures from shrinking due to a significant decrease in capillary forces during the drying process.

Brunauer–Emmett–Teller (BET) measurements were used to determine the specific surface areas and pore size distributions of the CF, cast FFCNF aerogel film (CAF that is prepared by solution casting of FCNF dispersion followed by a similar solvent exchange and ambient drying process as the DAF), DAF and samples with the exchange solvents of propanol and hexane. The nitrogen adsorption/desorption isotherms demonstrate that the DAF has a reasonably high specific surface area of 96 m^2^ g^−1^ (Fig. 2b), and the pore size distribution ranges from 5 to 13 nm (Fig. 2c), which is consistent with the previous morphological results. The CF exhibits the adsorption curve of characteristics of dense film with a smooth and flat surface (Figs. 2d and S7), and the CAF exhibits a wider pore size distribution than that of the DAF, which is supported by the SEM results (Fig. 2e). When the solvents of FFCNF hydrogel were replaced with propanol or hexane, the resultant samples had a delaminated microstructure with no obvious and almost no pore structures (Fig. 2f, g). The reason for the former lies in the surface energy differences between the FCNF and the surrounded solvents being too high to prevent the contraction of the aerogel structures due to relatively large capillary forces during the ambient drying process, while for the latter is that the hexane failing to displace the entire water trapped in the mesopores. To investigate the influence of FCNF concentrations on the porosity and specific surface area of the DAF, the solid contents of FCNF in the DAF were tailored. Three different FCNF concentrations, namely 0.5%, 1.0%, and 1.5%, were utilized, corresponding to DAF_0.5_, DAF_1.0_, and DAF_1.5_, respectively. The pore size distribution of DAF widened (Fig. S8) with the solid concentrations of FCNF increasing, while the specific surface area and pore volume increased and then reduced (Fig. S9). The DAF has a relatively large specific surface area and pore volume in the FFCNF-AF_1.0_.

### Mechanical, Optical and Thermal Properties of the DAF

**Fig. 3:**
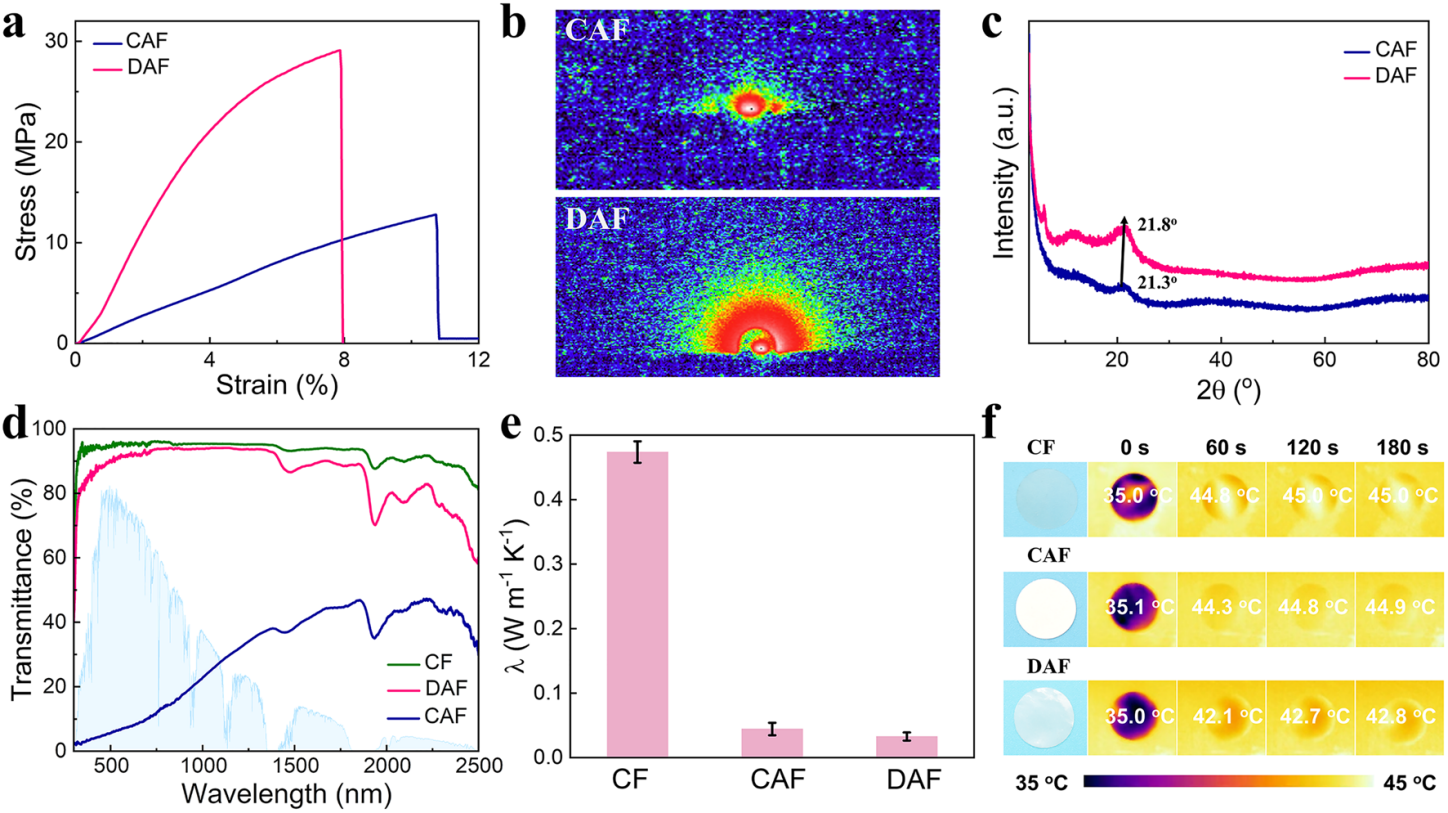
Mechanical, optical and thermal properties of the DAF. **a–c** Stress–strain curves, 2D SAXS patterns, and XRD patterns of CAF and DAF. **d, e** UV–vis-NIR full spectra and thermal conductivity of CF, CAF and DAF. **f** Thermographic images of CF, CAF and DAF on a 50 °C hot stage

The mechanical performance of aerogel films was further studied. The stress–strain curves of the CF, CAF and DAF are measured (Fig. 3a). The mechanical strength and Young's modulus of the DAF are 28.7 and 4.09 MPa, respectively, higher than those of CAF (12.8 and 1.2 Mpa) (Figs. S10 and S11). This is attributed to the presence of high-density hydrogen bonds and ordering delaminated stacking structures within the DAF, as confirmed by the small angle X-ray scattering (SAXS) with an anisotropic scattering in comparison with the CAF (Figs. 3b and S12). X-ray diffraction (XRD) analysis was used to characterize the interlamellar pore structures of the delaminated stacked DAF (Fig. 3c). The diffraction peak of FFCNF-AF at 2θ of 21.8° is larger than that of CAF (21.3°) indicating the formation of an interlamellar pore structure of the delaminated stacked DAF. This can be attributed to the formation of intense interactions of high-concentrated localized FCNF through hydrogen bonding during filtration.

Figure 3d displays the transmittances of the CF, CAF and DAF with a thickness of 100 µm from 300 to 2500 nm, which were measured by a UV–visible near-infrared spectrophotometer. The transmittance values for the CF, CAF, and DAF, respectively, are calculated as 92%, 29.4%, and 86.3% in the solar range (*T*_*solar*_ of 300–2500 nm); 95.1%, 7.5%, and 91.0% in the visible or luminous (*T*_*lum*_, 380–780 nm); and 92.7%, 36.6%, and 85.7% in the near-infrared range (*T*_*NIR*_, 780–2500 nm). The DAF exhibits a lower *T*_solar_ and *T*_*N*IR_ than those of CF due to the scattering in the near-infrared range caused by the intra-layer nanopore structures reducing solar thermal gain. The distinctive mesoporous structure of DAF plays a crucial role in minimizing light scattering, resulting in an enhanced transmittance within the 380–780 nm range compared to CAF. Notably, the *T*_*lum*_ of DAF decreases with increasing thickness, as illustrated in Fig. S13. The DAF exhibits exceptional thermal-insulating performance due to the formation of delaminated stacked structures and intra-layer nanopore structures that inhibit gas heat conduction. The thermal conductivity of the CF, CAF and DAF is measured as 474, 44, and 33 mW m^−1^ k^−1^ (Fig. 3e), respectively. Thermographic images of the CF, CAF, and DAF samples with similar sizes on a hot stage (surface temperature of 50 °C) were in situ measured by an infrared thermal imaging camera, demonstrating the conduction of heat flow from the hot stage to the interior of the sample over the periods of 0, 60, 120, and 180 s (Fig. 3f), respectively. The top-surface temperature of the DAF is 2.1–2.3 °C lower than those of the CF and CAF when these samples are placed on the hot stage and stabilized for 180 s, indicating that the DAF has superior thermal-insulating performance.

### Spectral Selectivity and Radiative Cooling Power of the DAF

**Fig. 4:**
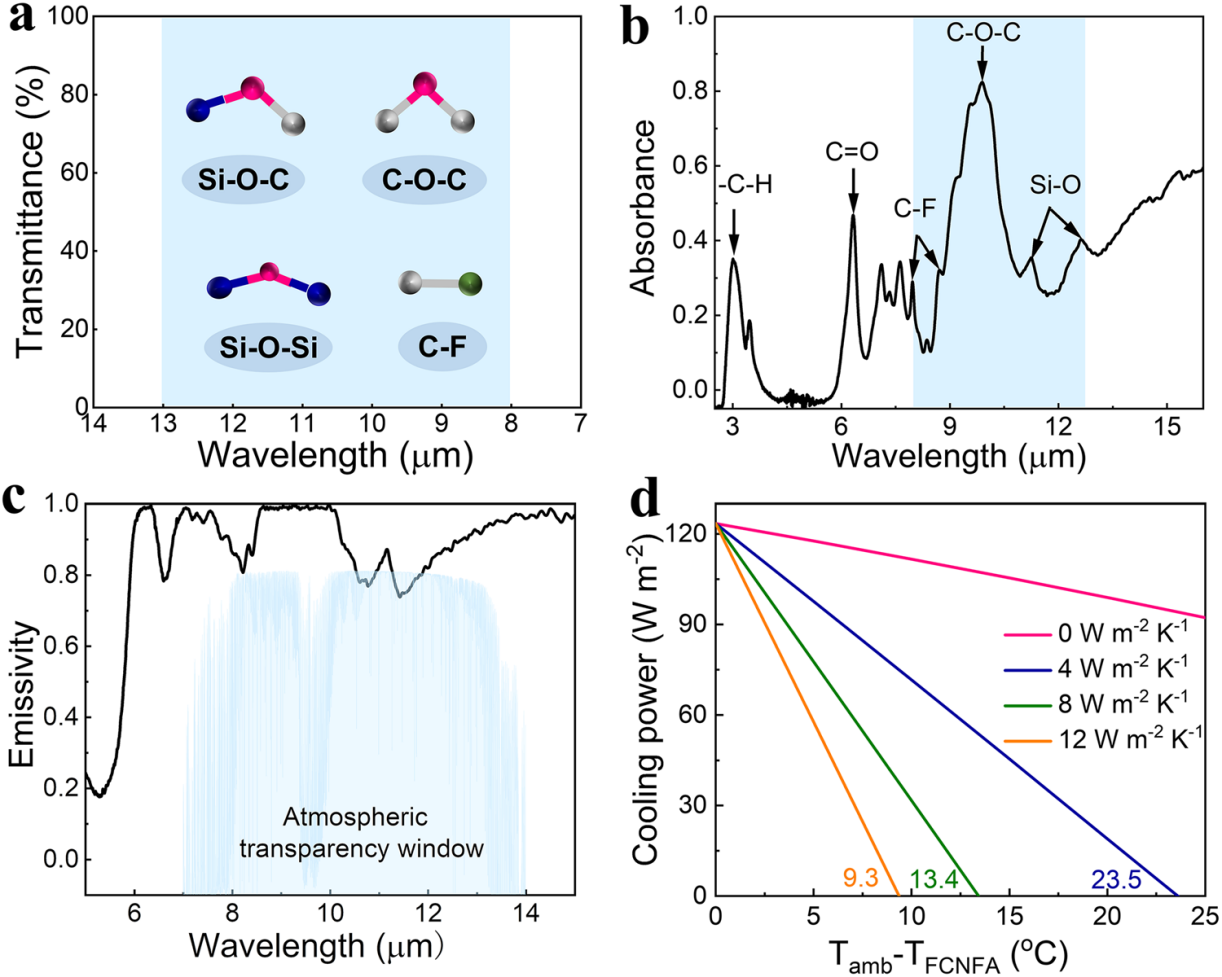
Spectral selectivity and radiative cooling power of the DAF. **a** Chemical groups of DAF and their infrared absorption peaks. **b–d** Absorbance, emission spectra, and calculated radiative power of DAF

The inherent features of DAF allow it for high-performance daylight radiative cooling. Figure 4a depicts the infrared absorption peaks of the functional groups of the DAF, mainly concluding the C-F (1120–1280 cm^−1^), Si–O-C (1005 cm^−1^), Si–O-Si (890 cm^−1^) and C–O–C (1100 cm^−1^) with the substantial stretching vibrations in the range of 8–13 µm. The molecular motion of DAF is increased when subjected to radiation in its unique absorption bands, resulting in severe stretching vibrations of interior atoms. As a sequence, electrons in their high energy band would simultaneously transit to the lower energy band, producing photons that can be radiated outward [48]. Finally, the DAF could emit heat in the mid-infrared range into outer space. The absorption spectra of the DAF reveal considerable infrared absorptions due to the stretching vibrations of C-F, Si–O, and C–O–C groups between the 8 and 13 µm coincidentally lying in the atmospheric transparency window (Fig. 4b), resulting in the high emissivity. Figure 4c shows that the average emissivity of the DAF in the atmospheric window is calculated as 90.1%, which is higher than that of the filtrated CNF aerogel film (Fig. S14). An energy balance model is developed to investigate the cooling effect of DAF. Considering the ambient temperature of 298.15 K and the non-radiative heat gain of 0, 4, 8, and 12 W m^−2^ K^−1^, the non-radiant 0 W m^−2^ K^−1^ represents the ideal adiabatic state and the non-radiant 4, 8, and 12 W m^−2^ K^−1^ represent the various parasitic heat loss circumstances. The decrease in the temperature of DAF under direct sunlight conditions is predicted by the theoretical model. Figure 4d shows that the radiative cooling power reaches the value of 123.5 W m^−2^ which is calculated from Eqs. (S3-S9) in Supporting Information. The maximum temperature decreases calculated from the non-radiative heat gains at 4, 8, and 12 W m^−2^ K^−1^ are 23.5, 13.4, and 9.3 K, respectively, indicating that the DAF has high radiative cooling performance.

### Solar-Thermal Dual-Regulation of the DAF

**Fig. 5:**
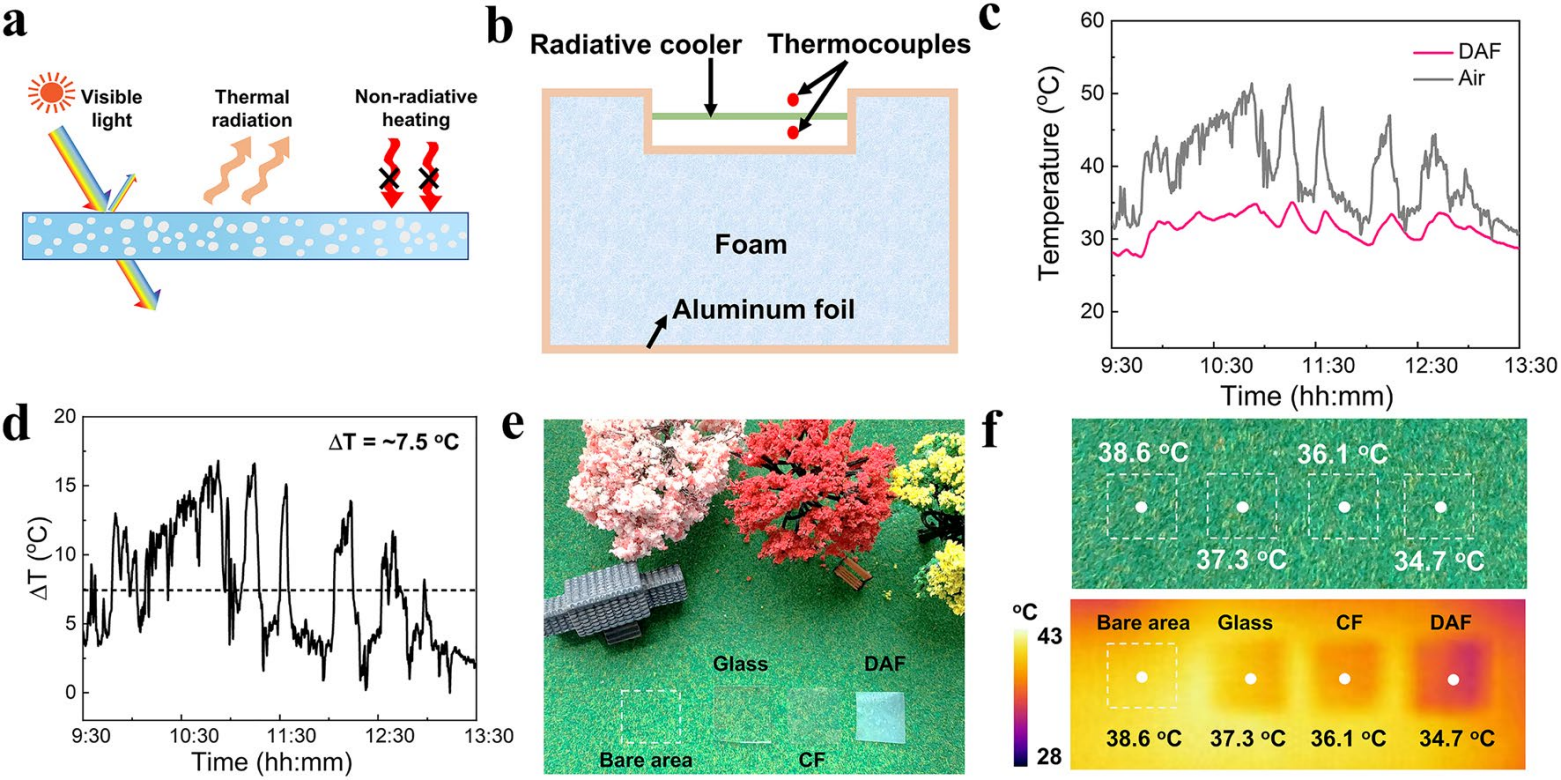
Solar-thermal dual-regulation of the DAF. **a** Schematic of the solar-thermal regulation of DAF. **b** Schematic of the apparatus for evaluating radiative cooling performance. **c** Temperature tracking of DAF and ambient air. **d** Temperature differences between the DAF and ambient air. **e** Temperatures monitoring for an artificial turf under direct sunlight conditions without and with the covering of glass, CF and DAF. **f** Thermographic images of the artificial turf after 2 h of sunlight exposure and removals of the covers

The presence of mesopores within the DAF could induce forward visible light scattering to fulfill indoor illumination (Fig. 5a). Meanwhile, the delaminated stacked and intra-layer mesopores structures provide the DAF with superior thermal-insulating performance that reduces the non-radiative heat gain. Furthermore, the chemical groups of C-F, C–O–C, Si–O-C, and Si–O-Si endow the DAF with a high emissivity of 90.1% in the range of 8–13 µm. Thus, the DAF produces excellent solar-thermal dual modulation performance to realize efficient indoor lighting and cooling. To validate the theoretical calculation, we designed a thermally insulated foam box composed of aluminum foil, thermocouples and polyethylene box (Fig. 5b) to evaluate the outdoor cooling performance of the experimental samples on the building roof on Oct. 3rd, 2022, Shanghai, China (121°2' east longitude, 31°11' north latitude). Figure 5c shows a 4-h comparison of the temperature varies of the DAF in comparison with the ambient air. The fluctuation of the atmospheric temperature is primarily caused by unpredictability like unexpected gusts. The temperature differences based on Fig. 5c are calculated to highlight the temperature varies between the DAF and the ambient air (Fig. 5d). The average temperature decrease of the DAF for 4 h under direct sunlight is about 7.5 °C, indicating the excellent radiative cooling performance that is comparable to the theoretical calculation value. The superior solar-thermal dual modulation capability of the DAF was further demonstrated using thermographic images of different objects after being exposed to direct sunlight. As shown in Fig. 5e, glass, CF and DAF were placed on artificial turf and exposed to the sunlight for 3 h. The cooling and insulating capabilities of the glass, CF and DAF, as quickly captured by the camera, are depicted in Fig. 5f. The temperature in the area covered by the DAF was the lowest at 34.7 °C, which is 2.6, 1.4, and 3.9 °C lower than that of the glass, the CF and the bare area, respectively.

### Durability of the DAF

**Fig. 6:**
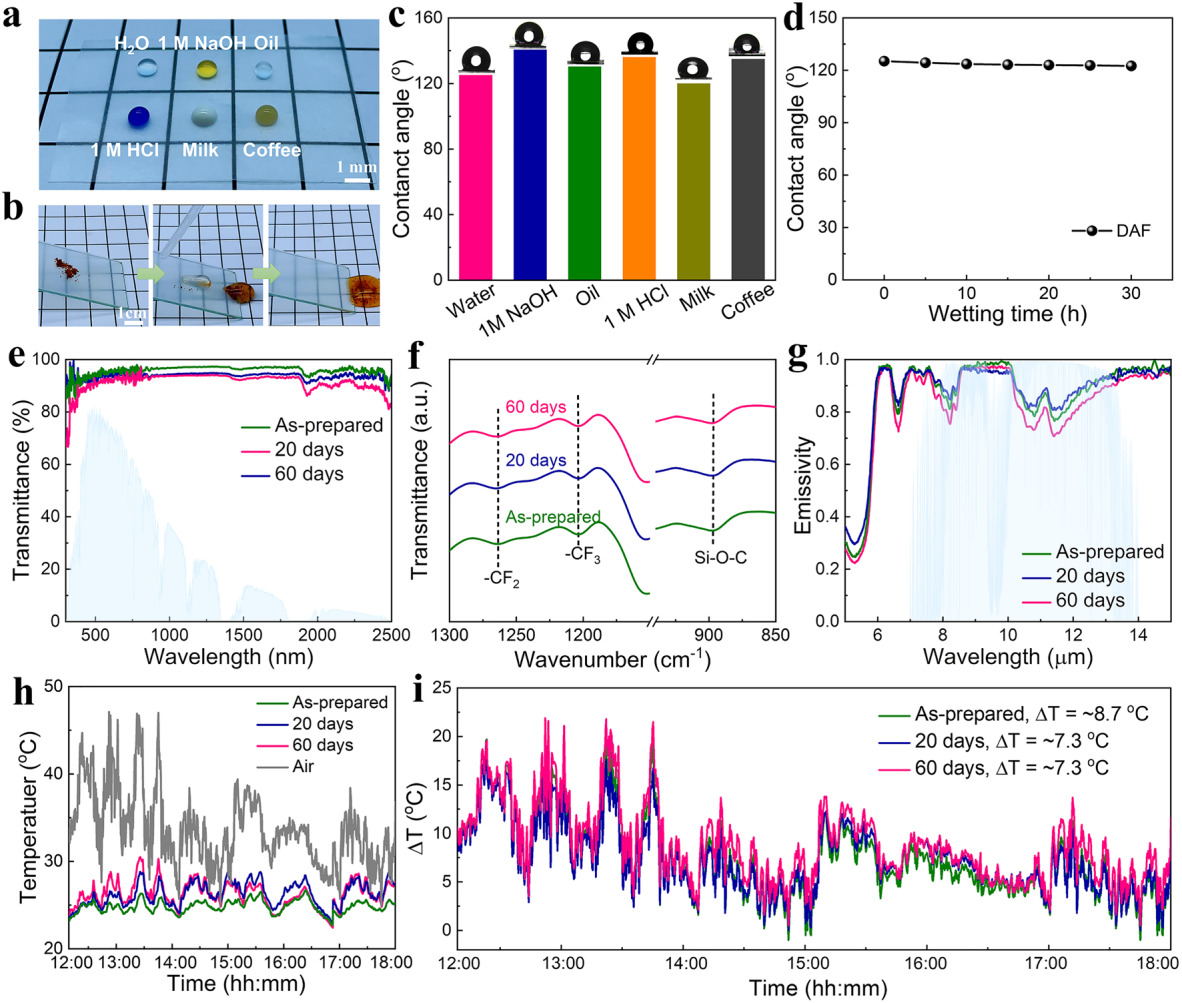
Durability of the DAF. **a** Photograph of casting the drops of water, 1 M NaOH, oil, 1 M HCl, milk and coffee on DAF. **b** Self-cleaning tests conducted on DAF using water flux to remove pigments. **c** Contact angles measured for water, 1 M NaOH, oil, 1 M HCl, milk and coffee on FFCNF-AF. **d** Changes of contact angles of DAF measured throughout 0–30 h. **e–g** UV–vis-NIR, FTIR, and emissivity spectra of DAF at various aging time. **h** Temperature tracking and **i** temperature differences between the spaces covered by DAF being aged after various time and the ambient air

The DAF also shows high hydrophobicity and excellent durability in complex outdoor high-temperature and humidity environments, attributed to the presence of abundant fluorine-containing groups that provide lower surface energy and resistance to UV irradiation. The DAF exhibits hydrophobicity with distinct oil repellency (Fig. 6a) and only produces approximately spherical droplets on the surface of DAF. Furthermore, with pigment as the model pollutant on the sample, flowing water could easily remove the pollutant, keeping the DAF dry and clean (Fig. 6b). The DAF shows the contact angles with water, 1 M NaOH, oil, 1 M HCl, milk, and coffee of 125°, 123°, 114°, 119°, 105°, and 108°, respectively (Fig. 6c). Figure 6d shows the changes in the water contact angles of the DAF from the initial to lasting for 30 h. The contact angles of the DAF are stable within 30 h, while that of the filtrated CNF aerogel film decreases rapidly after 20 s and remains at 31.9° only after 60 s (Fig. S15), suggesting the stable hydrophobicity of the DAF. This is because the ethoxy groups in perfluorodecyltriethoxysilane (PFOTES) decompose easily in water to generate silanol, converting the hydrophilic CNF surface into dehydrated Si–O-C covalent connections. Furthermore, the −CF_3_, −CF_2_, and Si–O-Si groups are highly hydrophobic with low surface energy.

Durability is a critical performance metric for high-performance solar-thermal controlled energy-saving windows. Thus, the aging test was used to evaluate the environmental stability of the DAF. After being exposed outdoors for 20 and 60 days, the DAF did not appear blistering, peeling, cracking, or discoloration, and its transmittance in the range of 380–790 nm remains above 85% (Fig. 6e). Figure 6f shows that the DAF has distinct absorption peaks both before and after aging due to the presence of abundant fluorine-containing groups, which still exhibit the characteristic stretching vibrations of −CF_2_ and −CF_3_ at 1255 and 1196 cm^−1^, respectively, as well as the absorption peaks of Si–O-C at 890 cm^−1^. The emissivity spectrum of DAF shows a slight fluctuation with the increasing aging time (Fig. 6g), which is due to the minimal yellowing effect after UV irradiation during the aging process. The cooling curves and temperature differences of DAF within 6 h before and after aging for 20 days and 60 days were tested on the roof (Oct. 14th, 2022, Shanghai, China), as shown in Fig. 6h. The average temperature difference between the as-prepared DAF and after being aged for 20 and 60 days is 8.7, 7.3 and 7.3 °C, respectively (Fig. 6i), demonstrating that the DAF has an outstanding cooling capability after long-term aging, further confirming its excellent durability for practical applications.

## Conclusions

In summary, we have successfully fabricated a highly porous yet transparent and mechanically flexible DAF via the filtration-induced delaminated gelation and ambient drying strategy. The strong intra-plane and relatively weak inter-layer hydrogen bonding maintain the structural stability of the delaminated and intra-layer nanopore structure within the DAF after the removal of non-polar solvents in ambient pressure conditions. The resulting DAF exhibits an impressive mechanical strength of 28.7 Mpa and shows excellent resistance to complex deformations, thanks to the high-density hydrogel bonding within the intra-layer nanofibers and between the delaminated stacked structures. The presence of mesoporous structures within the aerogel film contributes to its low thermal conductivity of 33 mW m^−1^ K^−1^. Additionally, the DAF demonstrates a high visible-light transmittance of 91.0%, and notably, the abundance of C–O–C, C-F, Si–O-Si, and Si–O-C groups in the DAF results in a high selective emissivity of 90.1% within the atmospheric transparent window. Furthermore, the DAF exhibits remarkable hydrophobicity and durability due to the presence of fluorine-containing groups, which contribute to its lower surface energy and UV resistance. By serving as a solar-thermal regulation energy-saving window, the DAF not only provides effective indoor lighting but also facilitates indoor radiative thermoregulation. In comparison with traditional transparent glass, the DAF achieves superior cooling performance, with a temperature decrease of approximately 2.6 °C under direct sunlight.

## Supplementary Information

Below is the link to the electronic supplementary material.

Supplementary file1 (PDF 946 KB)

## References

[1] K. Amasyali, N.M. El-Gohary, A review of data-driven building energy consumption prediction studies. Renew. Sustain. Energy Rev. **81**, 1192–1205 (2018). 10.1016/j.rser.2017.04.095

[2] L. Pérez-Lombard, J. Ortiz, C. Pout, A review on buildings energy consumption information. Energy Build. **40**, 394–398 (2008). 10.1016/j.enbuild.2007.03.007

[3] W. Chung, Review of building energy-use performance benchmarking methodologies. Appl. Energy **88**, 1470–1479 (2011). 10.1016/j.apenergy.2010.11.022

[4] E. Abraham, V. Cherpak, B. Senyuk, J.B. ten Hove, T. Lee et al., Highly transparent silanized cellulose aerogels for boosting energy efficiency of glazing in buildings. Nat. Energy **8**, 381–396 (2023). 10.1038/s41560-023-01226-7

[5] L. Zhao, X. Lee, R.B. Smith, K. Oleson, Strong contributions of local background climate to urban heat islands. Nature **511**, 216–219 (2014). 10.1038/nature13462 25008529 10.1038/nature13462

[6] S. Wang, Y. Zhou, T. Jiang, R. Yang, G. Tan et al., Thermochromic smart windows with highly regulated radiative cooling and solar transmission. Nano Energy **89**, 106440 (2021). 10.1016/j.nanoen.2021.106440

[7] B. Yu, Y. Wang, Y. Zhang, Z. Zhang, Self-supporting nanoporous copper film with high porosity and broadband light absorption for efficient solar steam generation. Nano-Micro Lett. **15**, 94 (2023). 10.1007/s40820-023-01063-z 10.1007/s40820-023-01063-z PMC10086088 37037910

[8] L. Cai, A.Y. Song, W. Li, P.-C. Hsu, D. Lin et al., Spectrally selective nanocomposite textile for outdoor personal cooling. Adv. Mater. **30**, e1802152 (2018). 10.1002/adma.201802152 30015999 10.1002/adma.201802152

[9] A.P. Raman, M. Abou Anoma, L. Zhu, E. Rephaeli, S. Fan, Passive radiative cooling below ambient air temperature under direct sunlight. Nature **515**, 540–544 (2014). 10.1038/nature13883 25428501 10.1038/nature13883

[10] P.-C. Hsu, A.Y. Song, P.B. Catrysse, C. Liu, Y. Peng et al., Radiative human body cooling by nanoporous polyethylene textile. Science **353**, 1019–1023 (2016). 10.1126/science.aaf5471 27701110 10.1126/science.aaf5471

[11] A. Leroy, B. Bhatia, C. Kelsall, A. Castillejo-Cuberos et al., High-performance subambient radiative cooling enabled by optically selective and thermally insulating polyethylene aerogel. Sci. Adv. **5**, eaat9480 (2019). 10.1126/sciadv.aat9480 31692957 10.1126/sciadv.aat9480 PMC6821464

[12] N.N. Shi, C.C. Tsai, F. Camino, G.D. Bernard, N. Yu et al., Thermal physiology. Keeping cool: enhanced optical reflection and radiative heat dissipation in saharan silver ants. Science **349**, 298–301 (2015). 10.1126/science.aab3564 26089358 10.1126/science.aab3564

[13] Q. Wu, Y. Cui, G. Xia, J. Yang, S. Du et al., Passive daytime radiative cooling coatings with renewable self-cleaning functions. Chin. Chemical Lett. **35**, 108687 (2024). 10.1016/j.cclet.2023.108687

[14] C. Buratti, E. Moretti, Glazing systems with silica aerogel for energy savings in buildings. Appl. Energy **98**, 396–403 (2012). 10.1016/j.apenergy.2012.03.062

[15] Q. Liu, A.W. Frazier, X. Zhao, J.A. De La Cruz, A.J. Hess et al., Flexible transparent aerogels as window retrofitting films and optical elements with tunable birefringence. Nano Energy **48**, 266–274 (2018). 10.1016/j.nanoen.2018.03.029

[16] R.C. Walker, A.P. Hyer, H. Guo, J.K. Ferri, Silica aerogel synthesis/process–property predictions by machine learning. Chem. Mater. **35**, 4897–4910 (2023). 10.1021/acs.chemmater.2c03459

[17] S. Luo, L. Peng, Y. Xie, X. Cao, X. Wang et al., Flexible large-area graphene films of 50–600 nm thickness with high carrier mobility. Nano-Micro Lett. **15**, 61 (2023). 10.1007/s40820-023-01032-6 10.1007/s40820-023-01032-6 PMC9984600 36867262

[18] Z. Jiao, W. Huyan, F. Yang, J. Yao, R. Tan et al., Achieving ultra-wideband and elevated temperature electromagnetic wave absorption via constructing lightweight porous rigid structure. Nano-Micro Lett. **14**, 173 (2022). 10.1007/s40820-022-00904-7 10.1007/s40820-022-00904-7 PMC9399338 35999287

[19] O.A. Tafreshi, Z. Saadatnia, S. Ghaffari-Mosanenzadeh, T. Chen, S. Kiddell et al., Flexible and shape-configurable PI composite aerogel films with tunable dielectric properties. Compos. Commun. **34**, 101274 (2022). 10.1016/j.coco.2022.101274

[20] X. Yu, X. Ren, X. Wang, G.H. Tang, M. Du, A high thermal stability core–shell aerogel structure for high-temperature solar thermal conversion. Compos. Commun. **37**, 101440 (2023). 10.1016/j.coco.2022.101440

[21] X. Li, H. He, Q. Liu, C. Zhao, H. Chen, Fabrication and property of hydrophobic polyvinyl alcohol/clay aerogel via irradiation-crosslinking and ambient-drying. Compos. Commun. **36**, 101359 (2022). 10.1016/j.coco.2022.101359

[22] L. Jian, G. Wang, X. Liu, H. Ma, Unveiling an S-scheme F-Co3O4@Bi2WO6 heterojunction for robust water purification. eScience (2023). 10.1016/j.esci.2023.100206

[23] E. Rephaeli, A. Raman, S. Fan, Ultrabroadband photonic structures to achieve high-performance daytime radiative cooling. Nano Lett. **13**, 1457–1461 (2013). 10.1021/nl4004283 23461597 10.1021/nl4004283

[24] Z. Chen, L. Zhu, A. Raman, S. Fan, Radiative cooling to deep sub-freezing temperatures through a 24-h day–night cycle. Nat. Commun. **7**, 13729 (2016). 10.1038/ncomms13729 27959339 10.1038/ncomms13729 PMC5159822

[25] K. Xu, Y. Wang, B. Zhang, C. Zhang, T. Liu, Stretchable and self-healing polyvinyl alcohol/cellulose nanofiber nanocomposite hydrogels for strain sensors with high sensitivity and linearity. Compos. Commun. **24**, 100677 (2021). 10.1016/j.coco.2021.100677

[26] R. Zhao, E. Songfeng, D. Ning, Q. Ma, B. Geng et al., Strengthening and toughening of TEMPO-oxidized cellulose nanofibers/polymers composite films based on hydrogen bonding interactions. Compos. Commun. **35**, 101322 (2022). 10.1016/j.coco.2022.101322

[27] M. He, M.K. Alam, H. Liu, M. Zheng, J. Zhao et al., Textile waste derived cellulose based composite aerogel for efficient solar steam generation. Compos. Commun. **28**, 100936 (2021). 10.1016/j.coco.2021.100936

[28] J. Wu, M. Zhang, M. Su, Y. Zhang, J. Liang et al., Robust and flexible multimaterial aerogel fabric toward outdoor passive heating. Adv. Fiber Mater. **4**, 1545–1555 (2022). 10.1007/s42765-022-00188-x

[29] T. Xue, C. Zhu, X. Feng, Q. Wali, W. Fan et al., Polyimide aerogel fibers with controllable porous microstructure for super-thermal insulation under extreme environments. Adv. Fiber Mater. **4**, 1118–1128 (2022). 10.1007/s42765-022-00145-8

[30] P.S. Weiss, How do we assess the impact of nanoscience and nanotechnology? ACS Nano **15**, 1–2 (2021). 10.1021/acsnano.1c00391 33498108 10.1021/acsnano.1c00391

[31] S. Tang, M. Ma, X. Zhang, X. Zhao, J. Fan et al., Covalent cross-links enable the formation of ambient-dried biomass aerogels through the activation of a triazine derivative for energy storage and generation. Adv. Funct. Mater. **32**, 2205417 (2022). 10.1002/adfm.202205417

[32] H. Françon, Z. Wang, A. Marais, K. Mystek, A. Piper et al., Ambient-dried, 3D-printable and electrically conducting cellulose nanofiber aerogels by inclusion of functional polymers. Adv. Funct. Mater. **30**, 1909383 (2020). 10.1002/adfm.201909383

[33] Z. Ye, C. Hu, J. Wang, H. Liu, L. Li et al., Burst of hopping trafficking correlated reversible dynamic interactions between lipid droplets and mitochondria under starvation. Exploration **3**, 20230002 (2023). 10.1002/EXP.20230002 37933279 10.1002/EXP.20230002 PMC10582609

[34] L. Wang, Y. Song, L. Li, L. Tao, M. Yan et al., Development of robust perovskite single crystal radiation detectors with high spectral resolution through synergetic trap deactivation and self-healing. InfoMat **5**, e12461 (2023). 10.1002/inf2.12461

[35] J. Yang, X. Shen, W. Yang, J.-K. Kim, Templating strategies for 3D-structured thermally conductive composites: recent advances and thermal energy applications. Prog. Mater. Sci. **133**, 101054 (2023). 10.1016/j.pmatsci.2022.101054

[36] R.J. Moon, A. Martini, J. Nairn, J. Simonsen, J. Youngblood, Cellulose nanomaterials review: structure, properties and nanocomposites. Chem. Soc. Rev. **40**, 3941–3994 (2011). 10.1039/C0CS00108B 21566801 10.1039/c0cs00108b

[37] X. Han, Z. Wang, L. Ding, L. Chen, F. Wang et al., Water molecule-induced hydrogen bonding between cellulose nanofibers toward highly strong and tough materials from wood aerogel. Chin. Chemical Lett. **32**, 3105–3108 (2021). 10.1016/j.cclet.2021.03.044

[38] T. Xue, Y. Yang, D. Yu, Q. Wali, Z. Wang et al., 3D printed integrated gradient-conductive MXene/CNT/polyimide aerogel frames for electromagnetic interference shielding with ultra-low reflection. Nano-Micro Lett. **15**, 45 (2023). 10.1007/s40820-023-01017-5 10.1007/s40820-023-01017-5 PMC9908813 36752927

[39] Y. Deng, Y. Yang, Y. Xiao, H.-L. Xie, R. Lan et al., Ultrafast switchable passive radiative cooling smart windows with synergistic optical modulation. Adv. Funct. Mater. **33**, 2301319 (2023). 10.1002/adfm.202301319

[40] H. Lai, Z. Chen, H. Zhuo, Y. Hu, X. Zhao et al., Defect reduction to enhance the mechanical strength of nanocellulose carbon aerogel. Chin. Chemical Lett. **35**, 108331 (2024). 10.1016/j.cclet.2023.108331

[41] J. Nemoto, T. Saito, A. Isogai, Simple freeze-drying procedure for producing nanocellulose aerogel-containing, high-performance air filters. ACS Appl. Mater. Interfaces **7**, 19809–19815 (2015). 10.1021/acsami.5b05841 26301859 10.1021/acsami.5b05841

[42] B. Wicklein, A. Kocjan, G. Salazar-Alvarez, F. Carosio, G. Camino et al., Thermally insulating and fire-retardant lightweight anisotropic foams based on nanocellulose and graphene oxide. Nat. Nanotechnol. **10**, 277–283 (2015). 10.1038/nnano.2014.248 25362476 10.1038/nnano.2014.248

[43] R. Zhang, B. Li, Y. Yang, N. Wu, Z. Sui et al., Ultralight aerogel sphere composed of nanocellulose-derived carbon nanofiber and graphene for excellent electromagnetic wave absorption. Nano Res. **16**, 7931–7940 (2023). 10.1007/s12274-023-5521-5

[44] M. Li, X. Chen, X. Li, J. Dong, X. Zhao et al., Controllable strong and ultralight aramid nanofiber-based aerogel fibers for thermal insulation applications. Adv. Fiber Mater. **4**, 1267–1277 (2022). 10.1007/s42765-022-00175-2

[45] X. Yang, E.D. Cranston, Chemically cross-linked cellulose nanocrystal aerogels with shape recovery and superabsorbent properties. Chem. Mater. **26**, 6016–6025 (2014). 10.1021/cm502873c

[46] W. Chen, Q. Zhang, K. Uetani, Q. Li, P. Lu et al., Absorption materials: sustainable carbon aerogels derived from nanofibrillated cellulose as high-performance absorption materials. Adv. Mater. Interfaces **3**, 9 (2016). 10.1002/admi.201600004

[47] S. Gamage, D. Banerjee, M.M. Alam, T. Hallberg, C. Åkerlind et al., Reflective and transparent cellulose-based passive radiative coolers. Cellulose **28**, 9383–9393 (2021). 10.1007/s10570-021-04112-1

[48] C. Cai, Z. Wei, C. Ding, B. Sun, W. Chen et al., Dynamically tunable all-weather daytime cellulose aerogel radiative supercooler for energy-saving building. Nano Lett. **22**, 4106–4114 (2022). 10.1021/acs.nanolett.2c00844 35510868 10.1021/acs.nanolett.2c00844

[49] K.-Y. Chan, X. Shen, J. Yang, K.-T. Lin, H. Venkatesan et al., Scalable anisotropic cooling aerogels by additive freeze-casting. Nat. Commun. **13**, 5553 (2022). 10.1038/s41467-022-33234-8 36138000 10.1038/s41467-022-33234-8 PMC9499976

