# Correction to: Highly Porous Yet Transparent Mechanically Flexible Aerogels Realizing Solar–Thermal Regulatory Cooling

**DOI:** 10.1007/s40820-024-01383-8

**Published:** 2024-03-21

**Authors:** Meng Lian, Wei Ding, Song Liu, Yufeng Wang, Tianyi Zhu, Yue-E. Miao, Chao Zhang, Tianxi Liu

**Affiliations:** 1 State Key Laboratory for Modification of Chemical Fibers and Polymer Materials, College of Materials Science and Engineering, Donghua University, Shanghai, 201620 People’s Republic of China; 2 Key Laboratory of Synthetic and Biological Colloids, Ministry of Education, School of Chemical and Material Engineering, Jiangnan University, Wuxi, 214122 People’s Republic of China

**Correction to:** Nano-Micro Lett. (2024) 16:131 10.1007/s40820-024-01356-x

Tianxi Liu was missed to be denoted as a corresponding author in the article. Both Chao Zhang and Tianxi Liu are the corresponding authors of this article.

The original article has been corrected.

