# Supplementary material for: Highly Porous Yet Transparent Mechanically Flexible Aerogels Realizing Solar-Thermal Regulatory Cooling
Source: Nanomicro Lett. 2024 Feb 26;16:131. doi: 10.1007/s40820-024-01356-x (PMC10897091; doi:10.1007/s40820-024-01356-x)
Supplement: Supplementary file 1 — Supplementary file1 (PDF 946 KB) [file 40820_2024_1356_MOESM1_ESM.pdf]

Supporting Information for

Highly Porous-Yet-Transparent Mechanically Flexible Aerogels Realizing Solar-Thermal Regulatory Cooling

Meng Lian[1], Wei Ding[1], Song Liu[1], Yufeng Wang[1], Tianyi Zhu[1], Yue-E Miao[1], Chao Zhang[1,]* and Tianxi Liu[2,]*

[1] State Key Laboratory for Modification of Chemical Fibers and Polymer Materials, College of Materials Science and Engineering, Donghua University, Shanghai 201620, P. R. China

[2] Key Laboratory of Synthetic and Biological Colloids, Ministry of Education, School of Chemical and Material Engineering, Jiangnan University, Wuxi 214122, P. R. China

*Corresponding authors. E-mail: czhang@dhu.edu.cn (Chao Zhang); txliu@jiangnan.edu.cn (Tianxi Liu)

S1 FTIR and XPS Characterizations

FTIR was utilized to analyze surface modification and characterize the molecular composition of FCNF. Figure S5a shows the infrared spectra of FCNF and CNF at 3800-600 cm^{-1}. The FCNF retains the characteristic absorption peak of CNF, and the broad absorption peak at 3395 cm^{-1} is attributed to the O-H stretching vibration in the molecular composition of CNF. The peak at 2874 and 1581 cm^{-1} are attributed to the C-H stretching vibration of CH_2 and the bending vibration of H-OH. The absorption peak at 1013 cm^{-1} is ascribed to C-O stretching vibration of the alcohol hydroxyl group of CNF [S1]. As shown in Fig. S5b, new absorption peaks appear in the infrared spectra at 1400-800 cm^{-1}, the peaks at 1255 and 1196 cm^{-1} are attributed to the characteristic stretching vibration of $-CF_2$ and $-CF_3$, respectively. In addition, the peaks at 1005 cm^{-1} and 890 cm^{-1} are attributed to Si-O-Si and Si-O-C [S2], respectively. XPS was used for qualitative and semi-quantitative analysis of FCNF and CNF. The peaks at 533.1 and 288.4 eV shown in Fig. S6a correspond to the oxygen and carbon in the CNF before modification. Three new peaks located at 689.5, 154.2, and 103 eV in the spectrum of F-CNF are attributed to F_{1s}, Si_{2s} and Si_{2p} [S3, S4]. As shown in Fig. S6b, a new peak appears at 290 eV in the FCNF spectrum and is attributed to the C-F bond in the PFOTES chain, indicating that PFOTES is grafted onto the surface of CNF. The restricted spectrum of C_{1s} was compared to the binding energy, which further confirmed the surface modification of CNF. Figure S6c, d shows the C_{1s} narrow scan spectra of CNF and FCNF, respectively. The peaks at 282.9, 284.6, and 286.1 eV are assigned to C1: C-C/C-H, C2: C-O and C3: O-C-O (three types of carbon bonds) [S5]. CNF is mainly a C-O bond (C2 type), while the new peaks of FCNF at 291.9 and 294.0 eV correspond to $-CF_2$ and $-CF_3$, respectively. This further confirms that PFOTES have been successfully grafted onto the surface of CNF.

S2 Calculation of Porosity

Porosity (P) of the CNFA is calculated by Eq. (S1):

$$P = \left(1 - \frac{\rho_0}{\rho}\right) * 100\% \qquad \text{(S1)}$$

where P is the porosity, ρ_0 is the apparent density of CNFA, and ρ is the volume density of polymer that is estimated from the average density of neat CNF of 1.6 g cm^{-3}.

S3 Calculation of T_{solar}, T_{lum}, and T_{IR}

The T_{solar} (300-2500 nm), T_{lum} (380-780 nm) , and T_{IR} (780-2500 nm) of the DAF are calculated by Eq. (S2):

$$T_{solar}, T_{lum}, T_{IR} = \frac{\int \varphi(\lambda) T(\lambda) d\lambda}{\int \varphi(\lambda) d\lambda} \qquad \text{(S2)}$$

$T(\lambda)$ denotes the recorded transmittance at a particular wavelength. For $T_{solar/IR}$, $\psi(\lambda)$ is the solar irradiance spectrum for AM 1.5. For T_{lum}, $\psi(\lambda)$ is the CIE "physiologically relevant" luminous efficiency function.

S4 Calculation of Radiative Cooling Power

Radiative cooling performance is influenced by solar irradiance and atmospheric downward thermal radiation. Meanwhile, heat can be transferred from the ambient surrounding to the radiative cooling sample through the thermal conduction and convection due to the temperature differences between the cooling sample and the ambient environment. The device meets the constraints of the power balance equation and the net cooling power ($P_{cool}(T)$) is calculated by Eq. (S3) [6]:

$$P_{cool}(\mathrm{T}) = P_{rad}(\mathrm{T}) - P_{atm}(T_{amb}) - P_{solar} - P_{nond+conv} \qquad \text{(S3)}$$

T is the surface temperature of cooling sample and T_{amb} is the ambient temperature. $P_{rad}(T)$ is the power radiated by the DAF, and $P_{atm}(T_{amb})$ is the absorbed atmospheric thermal radiation at T_{amb} . P_{solar} is the incident solar irradiation absorbed by the DAF, and $P_{nonrad+conv}$ is the power lost due to thermal convection and conduction. The net cooling power defined in Eq. (S3) could reach a high value by increasing the radiative power of DAF and reducing either the solar absorption or the parasitic heat gain [S7]. These parameters are calculated by the following Equations [S8, S9]:

$$P_{rad}(T) = A \int d\Omega \cos\theta \int_0^\infty d\lambda I_{BB}(T, \lambda) \varepsilon(\lambda, \theta) \qquad \text{(S4)}$$

$$P_{atm}(T_{amb}) = A \int d\Omega \cos\theta \int_0^\infty d\lambda I_{BB}(T_{amb}, \lambda) \varepsilon(\theta, \lambda) \varepsilon_{atm}(\lambda, \theta) \qquad \text{(S5)}$$

$$P_{solar} = A \int_0^\infty d\lambda \varepsilon(\lambda, \theta_{solar}) I_{AM1.5}(\lambda) \qquad \text{(S6)}$$

$$P_{nond+conv}(T, T_{amb}) = A \hbar_c (T_{amb} - T) \qquad \text{(S7)}$$

where A is the surface area of radiative cooler. The $\int d\,\Omega = 2\pi \int_0^{\pi/2} d\theta \sin\theta$ is the angular integral over a hemisphere. The $I_{BB}(\lambda, T) = \frac{2hc^2}{\lambda^5 e^{hc/\lambda kT} - 1}$ is the spectral radiance of a blackbody at the temperature (T). h is the Planck's constant, c is the speed of light in a vacuum and k is the Boltzmann constant. The $\varepsilon(\lambda, \theta)$ is the directional emissivity of the surface at wavelength λ. The $\varepsilon_{atm}(\lambda, \theta) = 1 - \tau(\lambda)^{1/con\theta}$ is the angle-dependent emissivity of the atmosphere, and $\tau(\lambda)$ is the atmospheric transmittance in the zenith direction. The $P_{rad}(T)$ and $P_{atm}(T_{amb})$ are determined by both spectral data of the DAF and the emissivity spectra of the atmosphere according to MODTRAN of the Mid-Latitude Summer Atmosphere Model. The solar illumination is represented by the AM1.5 spectrum ($I_{AM1.5}(\lambda)$) in Eq. (S6) [S10]. In Eq. (S7), $h_c = h_{cond} + h_{conv}$ is a combined nonradiative heat coefficient that captures the collective effect of conductive and convective heating, which is limited to a range between 0 and 12 W m^{-2} K^{-1}.

The average solar reflectance ($\bar{\rho}_{\mathrm{solar}}$) is defined as:

$$\bar{\rho}_{\mathrm{solar}} = \frac{\int_{0.3\,\mu m}^{2.5\,\mu m} \mathrm{I}_{\mathrm{solar}}(\lambda) * \rho_{\mathrm{solar}}(\lambda,\theta) d\lambda}{\int_{0.3\,\mu m}^{2.5\,\mu m} \mathrm{I}_{\mathrm{solar}}(\lambda) d\lambda} \quad \text{(S8)}$$

where λ is the wavelength of incident light in the range of 0.3-2.5 μm, $\mathrm{I}_{\mathrm{solar}}(\lambda)$ is the normalized ASTM G173 Global solar intensity spectrum, and $\rho_{\mathrm{solar}}(\lambda,\theta)$ is angular spectral reflectance of the surface. The average emittance ($\bar{\varepsilon}_{\mathrm{LWIR}}$) in the LWIR atmospheric transmittance window is defined as:

$$\bar{\varepsilon}_{\mathrm{LWIR}} = \frac{\int_{8\,\mu m}^{13\,\mu m} \mathrm{I}_{\mathrm{BB}}(\lambda,\mathrm{T}) * \varepsilon_{\mathrm{LWIR}}(\lambda,\theta) d\lambda}{\int_{8\,\mu m}^{13\,\mu m} \mathrm{I}_{\mathrm{bb}}(\lambda,\mathrm{T}) d\lambda} \quad \text{(S9)}$$

where $\mathrm{I}_{\mathrm{BB}}(\lambda,\mathrm{T})$ is the spectral intensity emitted by a blackbody and $\varepsilon_{\mathrm{LWIR}}(\lambda,\theta)$ is the surface's angular spectral thermal emittance in the range of 8-13 μm.

S5 Supplementary Figures

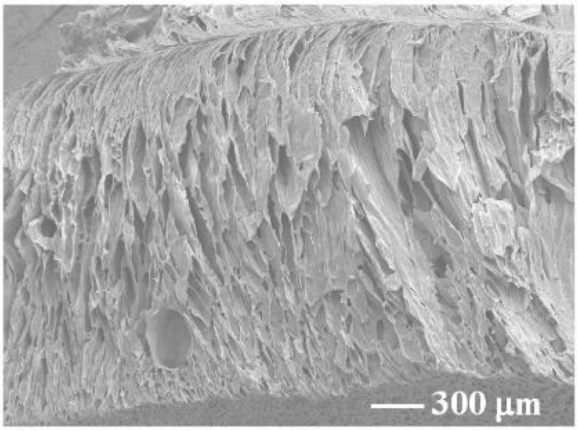

Fig. S1 SEM image of the freeze-dried FFCNF hydrogel sample taken halfway during the filtration

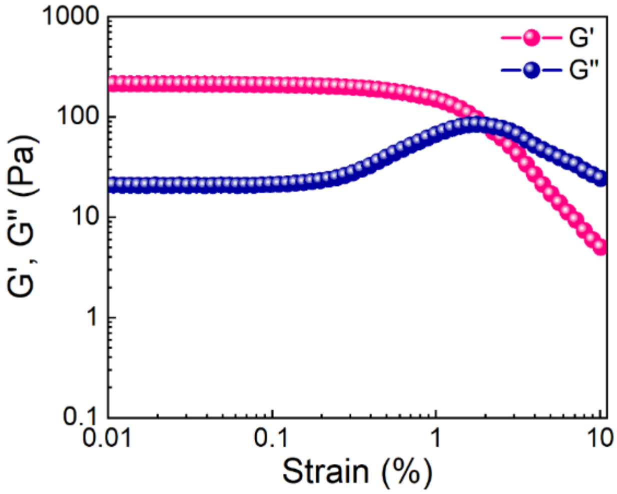

Fig. S2 Rheological property of the FFCNF hydrogel

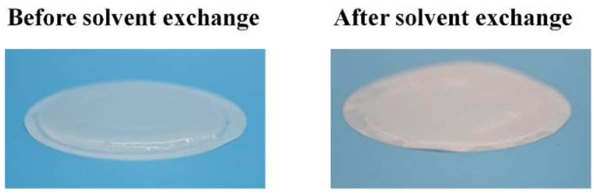

Fig. S3 Photographs of the FFCNF hydrogel before and after solvent exchange

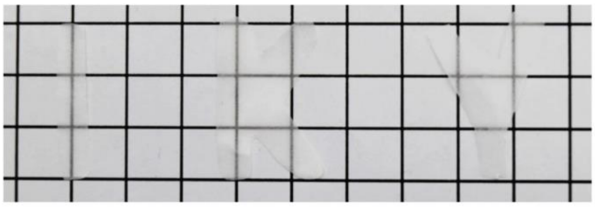

Fig. S4 Photograph of the DAF sample being cut into various shapes

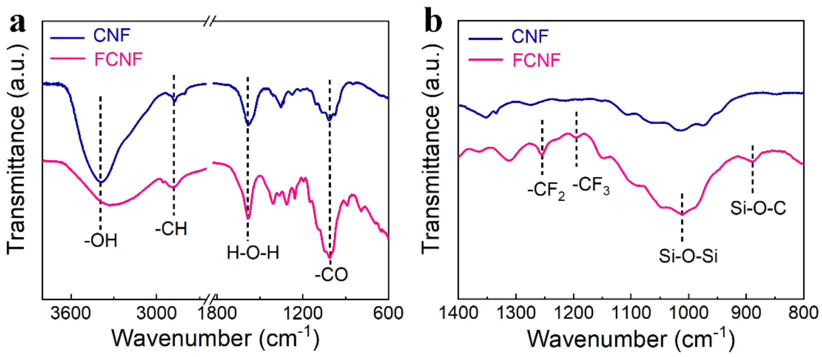

Fig. S5 FTIR spectra of the CNF and FCNF samples in the ranges of (**a**) 3800 to 600 cm^{-1}, and (**b**) 1400 to 800 cm^{-1}

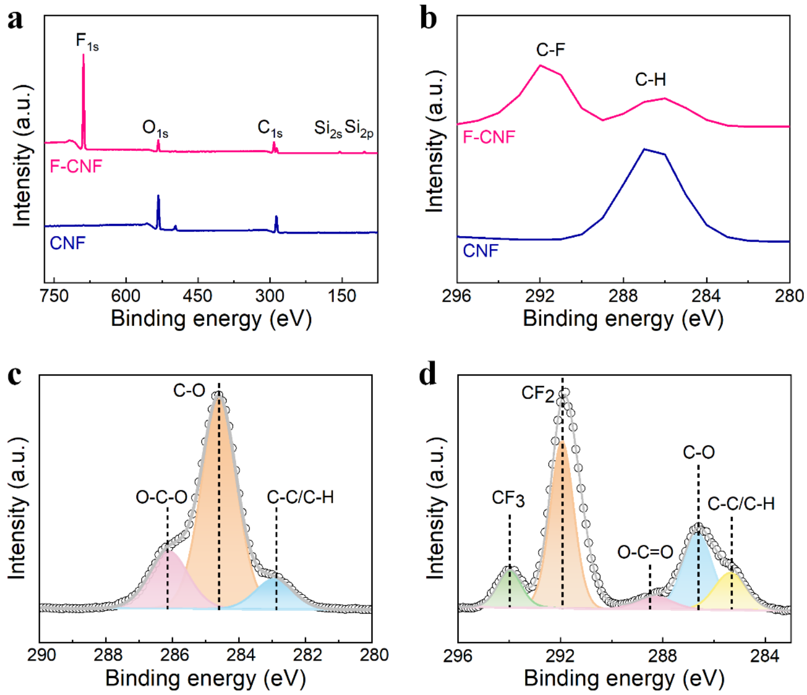

Fig. S6 (**a**) XPS survey spectra and (**b**) enlarged C 1s spectra of the CNF and FCNF. High-resolution C 1s spectra of (**c**) CNF and (**d**) FCNF

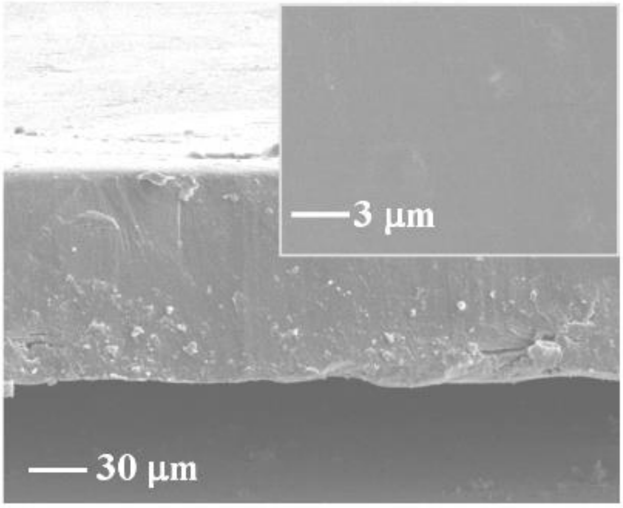

Fig. S7 Cross-section SEM image of the CF sample. Insert is the enlarged SEM image

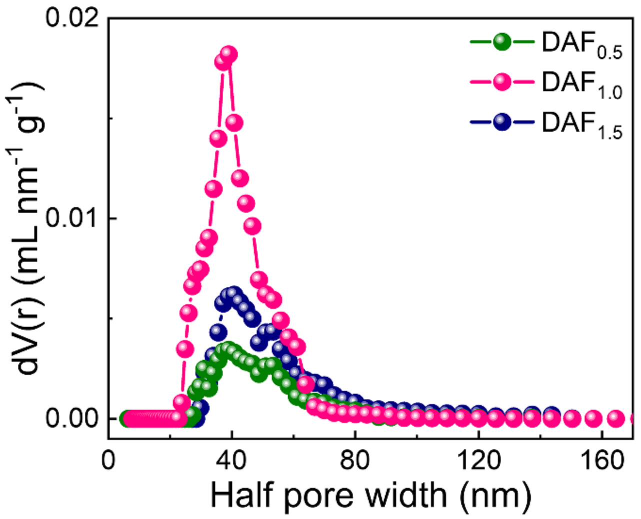

Fig. S8 Pore size distributions of the $DAF_{0.5}$, $DAF_{1.0}$ and $DAF_{1.5}$

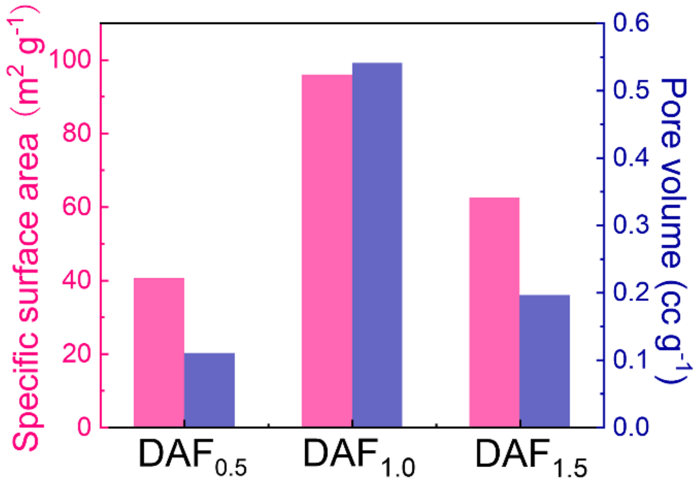

Fig. S9 Specific surface areas and pore volumes of the $DAF_{0.5}$, $DAF_{1.0}$ and $DAF_{1.5}$

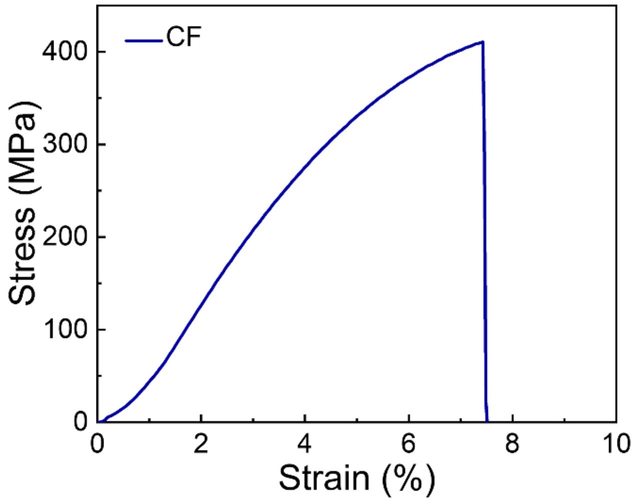

Fig. S10 Typical strain-stress curve of the CF

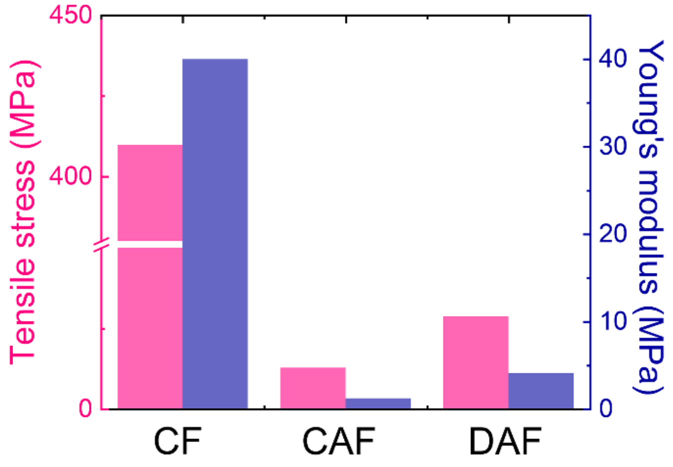

Fig. S11 Tensile stress and Young's modulus of the CF, CAF and DAF

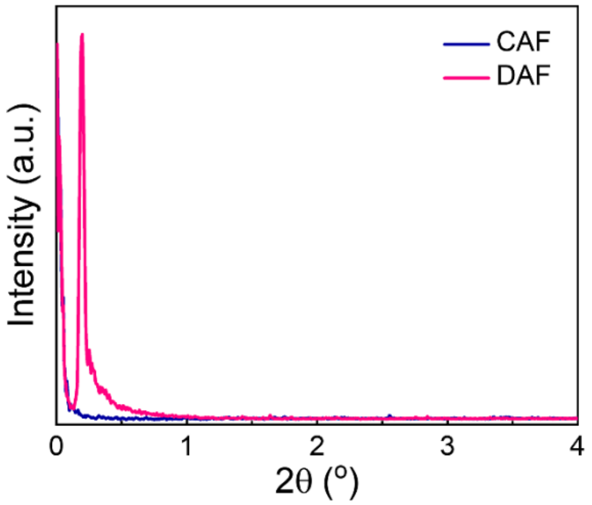

Fig. S12 Small-angle X-ray scattering patterns of the CAF and DAF

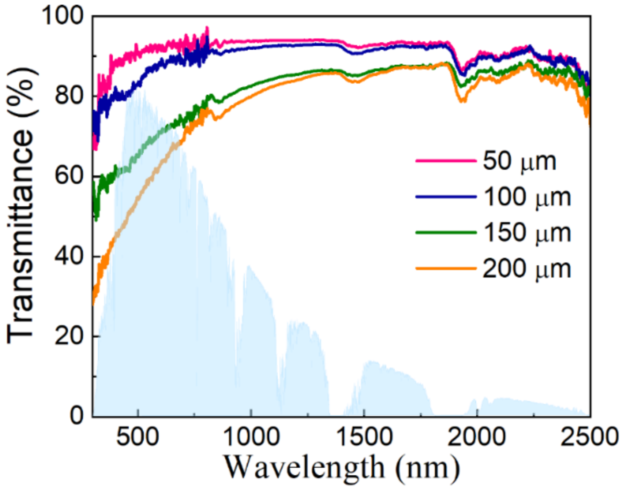

Fig. S13 UV-vis-NIR spectra of the DAF with the various thicknesses of 50, 100, 150, and 200 μm

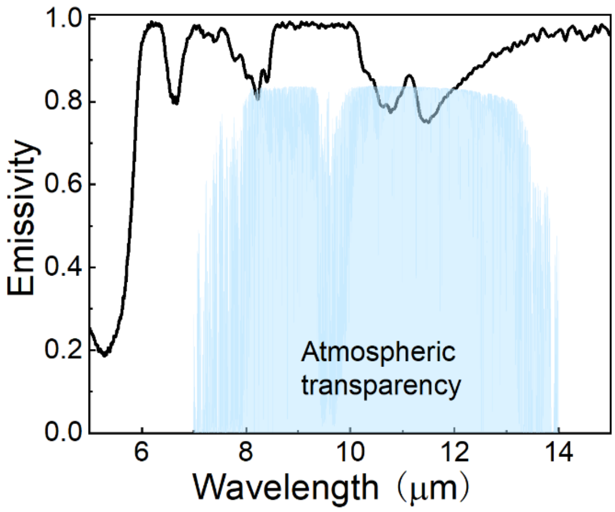

Fig. S14 Emissivity spectrum of the filtrated CNF aerogel film

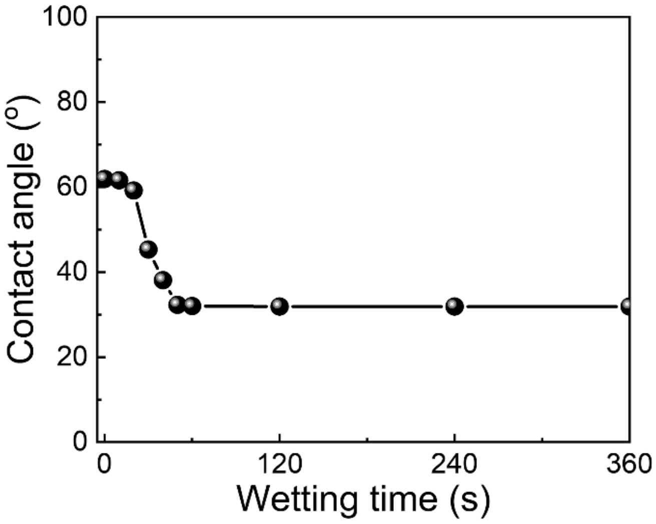

Fig. S15 Contact angles of the filtrated CNF aerogel film with the various wetting time

Supplementary References

[S1] B. Kaynak, M. Spoerk, A. Shirole, W. Ziegler, J. Sapkota, Polypropylene/cellulose composites for material extrusion additive manufacturing. Macromol. Mater. Eng. **303**, 8 (2018). https://doi.org/10.1002/mame.201800037

[S2] S. Nie, Q. Fu, X. Lin, C. Zhang, Y. Lu et al., Enhanced performance of a cellulose nanofibrils-based triboelectric nanogenerator by tuning the surface polarizability and hydrophobicity. Chem. Eng. J. **404**, 126512 (2021). https://doi.org/https://doi.org/10.1016/j.cej.2020.126512

[S3] L.S. Zhang, Y.H. Yu, G.P. Eyer, G.Q. Suo, L.A. Kozik et al., All-textile triboelectric generator compatible with traditional textile process. Adv. Mater. Technol. **1**, 8 (2016). https://doi.org/10.1002/admt.201600147

[S4] Y. Lu, S. Sathasivam, J.L. Song, C.R. Crick, C.J. Carmalt et al., Robust self-cleaning surfaces that function when exposed to either air or oil. Science **347**, 1132-1135 (2015). https://doi.org/10.1126/science.aaa0946

[S5] L.S. Johansson, T. Tammelin, J.M. Campbell, H. Setala, M. Osterberg, Experimental evidence on medium driven cellulose surface adaptation demonstrated using nanofibrillated cellulose. Soft Matter **7**, 10917-10924 (2011). https://doi.org/10.1039/c1sm06073b

[S6] X. Wang, X.H. Liu, Z.Y. Li, H.W. Zhang, Z.W. Yang et al., Scalable flexible hybrid membranes with photonic structures for daytime radiative cooling. Adv. Funct. Mater. **30**, 9 (2020). https://doi.org/10.1002/adfm.201907562

[S7] B.R. Sutherland, Charging up stationary energy storage. Joule **3**, 1-3 (2019). https://doi.org/10.1016/j.joule.2018.12.022

[S8] A.P. Raman, M.A. Anoma, L. Zhu, E. Rephaeli, S. Fan, Passive radiative cooling below ambient air temperature under direct sunlight. Nature **515**, 540-544 (2014). https://doi.org/10.1038/nature13883

[S9] E. Mu, Z. Wu, Z. Wu, X. Chen, Y. Liu et al., A novel self-powering ultrathin teg device based on micro/nano emitter for radiative cooling. Nano Energy **55**, 494-500 (2019). https://doi.org/10.1016/j.nanoen.2018.10.057

[S10] A. Leroy, B. Bhatia, C.C. Kelsall, A. Castillejo-Cuberos, H.M. Di Capua et al., High-performance subambient radiative cooling enabled by optically selective and thermally insulating polyethylene aerogel. Sci. Adv. **5**, 8 (2019). https://doi.org/10.1126/sciadv.aat9480
